# Amino acid response by Halofuginone in Cancer cells triggers autophagy through proteasome degradation of mTOR

**DOI:** 10.1186/s12964-019-0354-2

**Published:** 2019-05-02

**Authors:** Carlo Follo, Chiara Vidoni, Federica Morani, Alessandra Ferraresi, Christian Seca, Ciro Isidoro

**Affiliations:** 1 Laboratory of Molecular Pathology, Department of Health Sciences, Università del Piemonte Orientale “A. Avogadro”, Novara, Italy; 2 Dipartimento di Scienze della Salute, Università “A. Avogadro”, Via P. Solaroli 17, 28100 Novara, Italy; 3 Present address: Zuckerberg San Francisco General Hospital and Trauma Center, University of California San Francisco, San Francisco, CA 94110 USA

**Keywords:** Autophagy, Starvation, mTORC1, Lysosome, TFEB, Protein translation

## Abstract

**Background:**

In the event of amino acid starvation, the cell activates two main protective pathways: Amino Acid starvation Response (AAR), to inhibit global translation, and autophagy, to recover the essential substrates from degradation of redundant self-components. Whether and how AAR and autophagy (ATG) are cross-regulated and at which point the two regulatory pathways intersect remain unknown. Here, we provide experimental evidence that the *mammalian target of rapamycin* (mTOR) *complex 1* (mTORC1) specifically located at the lysosome level links the AAR with the autophagy pathway.

**Methods:**

As an inducer of the AAR, we used halofuginone (HF), an alkaloid that binds to the prolyl-tRNA synthetase thus mimicking the unavailability of proline (PRO). Induction of AAR was determined assessing the phosphorylation of the *eukaryotic translation initiation factor* (eIF) 2α. Autophagy was monitored by assessing the processing and accumulation of *microtubule-associated protein 1 light chain 3 isoform B* (LC3B) and *sequestosome-1* (p62/SQSTM1) levels. The activity of mTORC1 was monitored through assessment of the phosphorylation of mTOR, (rp)S6 and 4E-BP1. Global protein synthesis was determined by puromycin incorporation assay. mTORC1 presence on the membrane of the lysosomes was monitored by cell fractionation and mTOR expression was determined by immunoblotting.

**Results:**

In three different types of human cancer cells (thyroid cancer WRO cells, ovarian cancer OAW-42 cells, and breast cancer MCF-7 cells), HF induced both the AAR and the autophagy pathways time-dependently. In WRO cells, which showed the strongest induction of autophagy and of AAR, global protein synthesis was little if any affected. Consistently, 4E-BP1 and (rp)S6 were phosphorylated. Concomitantly, mTOR expression and activation declined along with its detachment from the lysosomes and its degradation by the proteasome, and with the nuclear translocation of *transcription factor EB* (TFEB), a transcription factor of many ATG genes. The extra supplementation of proline rescued all these effects.

**Conclusions:**

We demonstrate that the AAR and autophagy are mechanistically linked at the level of mTORC1, and that the lysosome is the central hub of the cross-talk between these two metabolic stress responses.

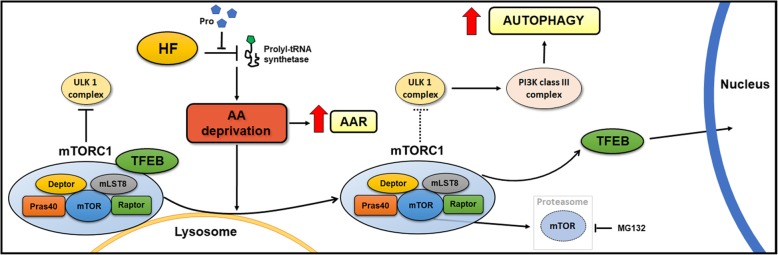

## Background

Mammalian cells have developed adaptive mechanisms to sense and counteract amino acid deprivation in order to maintain a homeostatic intracellular pool of amino acids [1]. According to the availability of amino acids, mammalian cells regulate protein synthesis and autophagy [2, 3]. Autophagy is a lysosomal degradation process through which the cell eliminates the redundant or damaged cytosolic material to recover the basic components to secure the macromolecular turnover and cell homeostasis. Autophagy promptly raises up when the cell is subjected to an adverse nutrient condition, such as in the case of amino acid deprivation [4].

A first sensor of amino acids levels is provided by the *general control nonderepressible 2 kinase* (GCN2) that detects the uncharged tRNAs resulting from the lack of amino acids (1, 5). In this situation, GCN2 phosphorylates the Serine 51 of the α-subunit of e*ukaryotic translation initiation factor* (eIF) 2α. Such phosphorylation causes a reduction in translation initiation and protein synthesis. Also, phosphorylated eIF2α promotes the translation of specific mRNAs containing in their 5′ leader unique upstream open reading frames, such as the *activating transcription factor 4* (ATF4) mRNA. In turn, ATF4 triggers the transcriptional *amino acid response* pathway (AAR) by inducing the expression of several target genes, including *activating transcription factor 3* (ATF3), *CEBP homologous protein* (CHOP) and *asparagines synthetase* (ASNS) [1, 5–7]. Of note, recent works indicate that the deprivation of different individual amino acids may trigger distinct AARs [1, 8].

A second sensor of amino acids levels is provided by the *mammalian target of rapamycin* (mTOR) *complex 1* (mTORC1). The complex includes mTOR, the *40 kDa Pro-rich Akt substrate* (PRAS40), the *mammalian lethal with SEC13 protein 8* (mLST8), the *DEP domain-containing mTOR-interacting protein* (DEPTOR) and the *regulatory-associated protein of mTOR* (RAPTOR) [3]. When active, mTORC1 promotes cell growth by stimulating the protein synthesis through the phosphorylation of the *eIF4E-binding protein 1* (4E-BP1) and of *p70S6 kinase 1* that in turn phosphorylates the *ribosomal protein S6* (S6). Particularly, the phosphorylation of Thr37/46, Thr70 and Ser65 in 4E-BP1 frees eIF4E that can then bind to eIF4G allowing the initiation of cap-dependent translation.

Moreover, active mTORC1 inhibits autophagy by phosphorylating the autophagy-related (ATG) proteins ATG13 and *Unc-51 Like Autophagy Activating Kinase 1* (ULK1). The activity of mTORC1 is regulated by several signals, including growth factors, cellular energy level, oxygen level and nutrients, particularly amino acids [3, 9, 10]. Upon amino acid deprivation, mTORC1 is inactivated with the resulting inhibition of protein synthesis and activation of autophagy. Subcellular control of mTORC1 by amino acids levels occurs via the Rag GTPases that are held on the membranes of the late endosomes/lysosomes (LEL) by the Ragulator (LAMTOR) complex. In presence of amino acids, the Rags positively regulate mTORC1 by recruiting the complex on the LEL membranes [11, 12].

Clearly, the AAR and the autophagy processes must be coordinated by the availability of amino acids. Whether and how these processes are cross-regulated and at which point the two regulatory pathways intersect remain unknown. Here, we investigated on these issues taking advantage of the molecular mechanism of action of the febrifugine-derivative halofuginone (HF). This drug was reported to mimic an AAR in Th17 lymphocytes by interfering with the utilization of proline [13–15]. Here, we show that in several cancer cell lines HF induces the AAR and concomitantly triggers the autophagy response by promoting the proteasome-mediated degradation of mTOR and the nuclear translocation of the autophagy transcription factor TFEB. An excess of proline could prevent all these events, proving that the unavailability of one single (particular) amino acid can trigger both the AAR and autophagy. Interestingly, we found that HF had a little impact on global protein synthesis and stimulated mTORC2 activity. Our data provide the first demonstration that the AAR and autophagy are mechanistically linked and suggest that the therapeutic properties of HF could be mediated by autophagy.

## Methods

### Reagents

Unless otherwise specified, culture media, antibiotics, antibodies and analytical grade chemicals were from Sigma-Aldrich Corp., St. Luis, MO, USA. Primary antibodies were obtained from the following sources: rabbit monoclonal anti-ATG7 (04–1055, EMD Millipore Corporation, Billerica, MA, USA), mouse monoclonal anti-eIF2α (2103, Cell Signaling Technology Inc., Danvers, MA, USA), rabbit monoclonal anti-phospho-eIF2α Ser 51 (3398, Cell Signaling Technology Inc.), mouse monoclonal anti-Golgin 97 (sc-59,820, Santa Cruz Biotechnology Inc., Dallas, TX, USA), mouse monoclonal anti-LAMP-1 (555,798, Becton, Dickinson and Company, New Jersey, NJ, USA), rabbit polyclonal anti-LC3B (L7543, Sigma-Aldrich Corp.), rabbit monoclonal anti-p62/SQSTM1 (D5E2) (8025, Cell Signaling Technology Inc.), rabbit polyclonal S6 ribosomal protein (5G10) (2217, Cell Signaling Technology Inc.), rabbit monoclonal anti-phospho-S6 ribosomal protein (Ser235/236) (4856, Cell Signaling Technology Inc.), rabbit monoclonal anti-mTOR (2983, Cell Signaling Technology Inc.), rabbit polyclonal anti-phospho-mTOR Ser 2448 (2971, Cell Signaling Technology Inc.), rabbit polyclonal anti-phospho-mTOR Ser 2481 (2974, Cell Signaling Technology Inc.), mouse monoclonal anti-β-Tubulin (T5293, Sigma-Aldrich Corp.), rabbit monoclonal anti-RAPTOR (2280, Cell Signaling Technology Inc.), rabbit polyclonal TFEB (4240, Cell Signaling Technology Inc.), Rabbit monoclonal anti-4E-BP1 (53H11) (9644, Cell Signaling), Rabbit monoclonal anti-phospho- 4E-BP1 Thr 37/46 (236B4) (2855, Cell Signaling) Rabbit polyclonal anti-phospho-Akt Ser 473 (9271, Cell Signaling), Rabbit monoclonal anti-Akt (pan) (11E7) (4685, Cell Signaling), Mouse monoclonal anti-β-actin clone AC-15 (A5441, Sigma-Aldrich), Mouse monoclonal anti-puromycin clone 12D10 (MABE343, Merck Millipore, Darmstadt, Germany). Secondary antibodies employed for immunoblotting were purchased from the following sources: Horse Radish Peroxidase-conjugated goat anti-mouse IgG (170–6516, Bio-Rad, Hercules, CA, USA), Horse Radish Peroxidase-conjugated goat anti-rabbit IgG (170–6515, Bio-Rad, Hercules, CA, USA). Secondary antibodies employed for immunofluorescence were purchased from the following sources: IRIS 2 goat anti-rabbit IgG (2WS-08, Cyanine Technologies S.p.A., Torino, Italy), IRIS 3 goat anti-mouse IgG (3WS-07, Cyanine Technologies S.p.A., Torino, Italy).

### Cell cultures and treatments

The following tumor-derived human cell lines available from the ATCC (Rockville, MD, USA) were used: WRO (thyroid carcinoma), MCF-7 (breast adenocarcinoma), and OAW-42 (ovarian carcinoma). The cells were cultured under standard conditions (37 °C, 5% CO2) in RPMI (WRO) or Minimum Essential Medium (MCF-7 and OAW-42). Media were supplemented with 10% fetal bovine serum (Lonza, Basel, Switzerland), 2 mM glutamine and 1% penicillin-streptomycin solution. Where indicated, cells were exposed to 100 nM halofuginone (HF) in presence or absence of 10 mM ammonium chloride (NH_4_^+^), or 30 μM chloroquine (CQ), or 10 μM MG132, or 35 μM cycloheximide (CHX) in complete culture medium or Earle’s Balanced Salt Solution (EBSS) for the indicated time. EBSS contains 1% glucose and is widely used as a culture medium to starve the cells of amino acids and serum growth factors (E2888). In rescue experiments, extra proline was added to WRO complete culture medium. Proline was supplied 10 times more concentrated than the original culture medium concentration (2 mM).

### siRNA and plasmid transfection

siRNA and plasmid transfections were performed following manufacturer’s protocols with Lipofectamine 2000 (Life Technologies Ltd., Paisley, UK). Treatments were performed 36 h after the transfection. siRNA sequences: control duplex siRNA 5′-AGG UAG UGU AAU CGC CUU GTT-3′; ATG7 siRNA 5′-GGG UUA UUA CUA CAA UGG UGT T-3′. The origin and use of the GFP-FYVE and GFP-LC3 plasmids have been reported previously [16–18].

### Immunoblotting

Cells were harvested in RIPA Buffer supplemented with protease inhibitor cocktail, and phosphatase inhibitors (sodium fluoride and sodium orthovanadate) and homogenized using an ultrasonic cell disruptor XL (Misonix, Farmingdale, NY, US). Protein concentration was assessed with a Bradford assay and equal amounts of protein (30 μg of total cell homogenates) were separated by SDS-PAGE and transferred onto PVDF membrane. After blocking with 5% non-fat milk (Santa Cruz Biotechnology Inc.), the filter was probed with designated primary and secondary antibodies, developed with the enhanced chemiluminescence method (PerkinElmer Inc., Waltham, MA, USA). Bands were imaged and subjected to densitometry using the VersaDOC Imaging System apparatus (Bio-Rad) equipped with the software Quantity One (Bio-Rad). Representative western blotting of at least three independent experiments are shown.

### Immunofluorescence

WRO cells were plated on coverslips at 25,000 cells/cm^2^ and let adhere 24 h before the designated treatments. The cells were then washed in PBS, fixed over night with 4% paraformaldehyde at 4 °C, permeabilized with 0.2% Triton X-100 in PBS for 10 min and processed for immunostaining with indicated primary antibodies and corresponding secondary antibodies. Images were captured with a Leica DMI6000 fluorescence microscope (Leica Microsystems AG, Wetzlad, DE) equipped with the software Leica Application Suite V. 3.8 (Leica Microsystems AG). Representative images of at least three independent experiments are shown.

### Subcellular fractionation

WRO cells were cultured in presence or absence of 100 nM halofuginone for 8 h, washed twice with ice-cold PBS and harvested in homogenization buffer (0.25 M sucrose, 2 mM Hepes Buffer, PBS) supplemented with protease inhibitor cocktail, and phosphatase inhibitors (sodium fluoride and sodium orthovanadate). Cell suspensions were homogenized using a 2 ml dounce tissue grinder and then centrifuged at 1000x g for 10 min at 4 °C in order to obtain post-nuclear supernatants (PNS). The PNS were loaded on 11 ml of 15 to 65% discontinuous sucrose gradient prepared in homogenization buffer and centrifuged at 20,0000x g for 16 h at 4 °C using a SW-41 swing rotor (Beckman Coulter, Inc., Brea, CA, USA). Twelve fractions (1 ml each) were collected from the top of the gradient and processed for immunoblotting analysis with the indicated antibodies.

### Puromycin incorporation assay

WRO cells were seeded on p35 petri at 40,000 cells/cm^2^ and let adhere 24 h before to perform the treatment. After exposure to 100 nM HF in presence/absence of 2 mM proline in complete medium or incubation with EBSS for the indicated time, the cell pellets were washed with PBS 1X, supplemented with 5 μg/ml puromycin (P7255, Sigma-Aldrich) and incubated at 37 °C for 10 min. The cell pellets were then washed with PBS1X and incubated at 37 °C for 5 min. Cells were lysed in RIPA Buffer supplemented with protease inhibitor cocktail and phosphatase inhibitors and the homogenates used for western blotting.

### Data analysis and statistics

All data were replicated at least three times in separate experiments. Densitometric analyses of immunoblot bands were performed with Quantity One (Bio-Rad laboratories) software. Differences between indicated protein ratios were analyzed by Student *t* test. A *p* value ≤ of 0.05 was considered significant. Immunofluorescence intensity density was determined with ImageJ 1.48v (http://imagej.nih.gov/ij/) software. GraphPad Prism was employed for statistical analysis (GraphPad Software Inc.).

## Results

### Halofuginone induces the amino acid response pathway in human cancer cell types

**Fig. 1:**
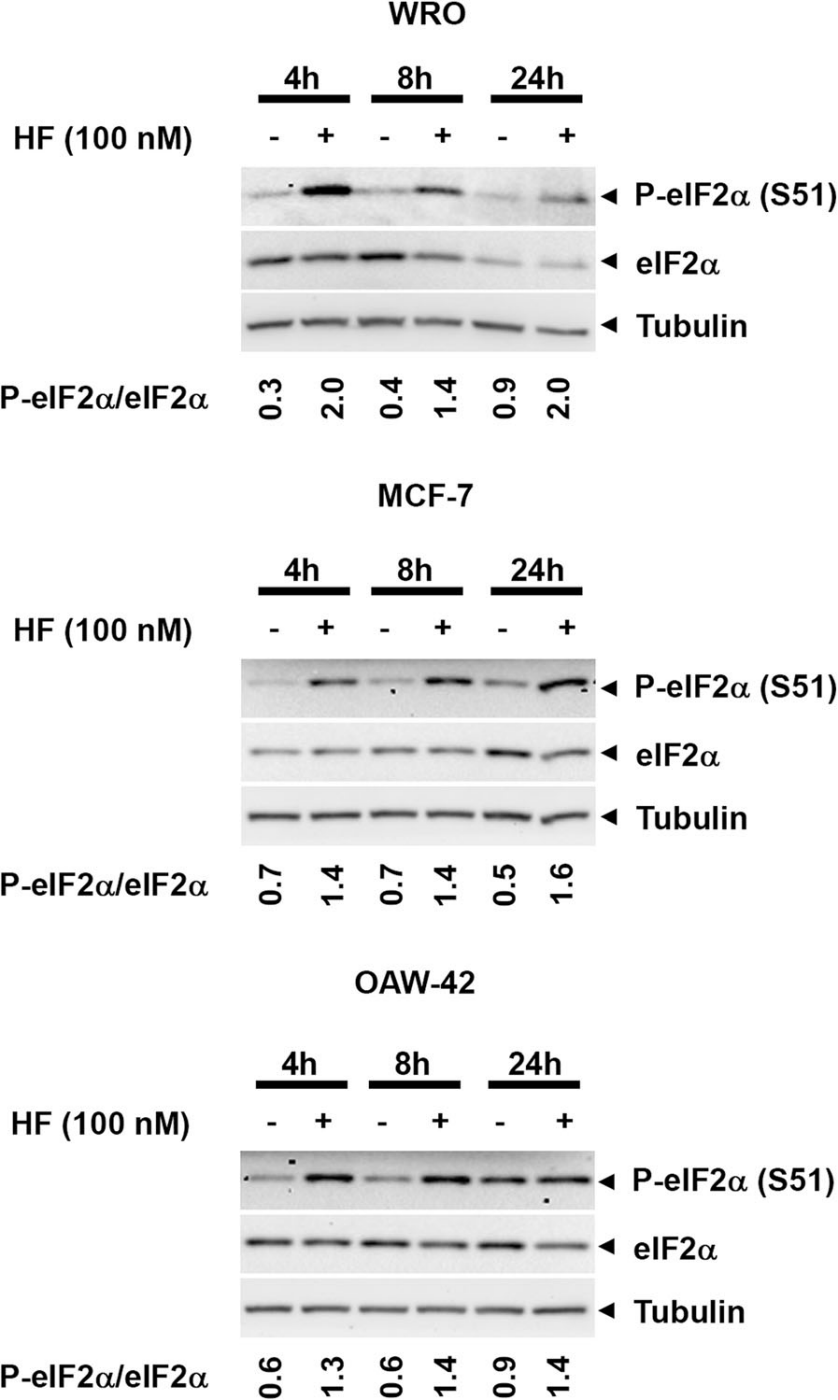
Halofuginone induces the amino acid starvation response (AAR) pathway. Thyroid cancer WRO cells, breast cancer MCF-7 cells, and ovarian cancer OAW-42 cells were exposed to 100 nM halofuginone (HF) for 4, 8 and 24 h. Total and phosphorylated (Ser 51) eIF2α protein levels were assessed by immunoblotting. As loading control, filters were stripped and probed with anti β-Tubulin as loading control. Representative immunoblots from three independent experiments are shown along with P-eIF2α/eIF2α band intensity ratios as index of AAR

In a first set of experiments, three different types of human cancer cells, namely thyroid cancer WRO cells, ovarian cancer OAW-42 cells, and breast cancer MCF-7 cells, were exposed to 100 nM HF and eIF2α phosphorylation, chosen to monitor the induction of the AAR, was assessed at 4, 8 and 24 h. Increased phosphorylation of eIF2α was clearly detectable in all the cell lines starting from 4 h of treatment with HF (Fig. 1). The level of phosphorylated eIF2α was differently modulated along the incubation time in the three different cell lines (Fig. 1). Our data confirm that HF induces the AAR pathway in all the investigated cancer cell lines.

### The amino acid starvation response induced by halofuginone is paralleled by up-regulation of basal autophagy

Next, we checked whether autophagy was induced along with the AAR by HF. Processing and accumulation of lipidated *microtubule-associated protein 1 light chain 3 isoform B* (LC3B) were assumed as an index of the autophagosomes present in the cells [19].

**Fig. 2:**
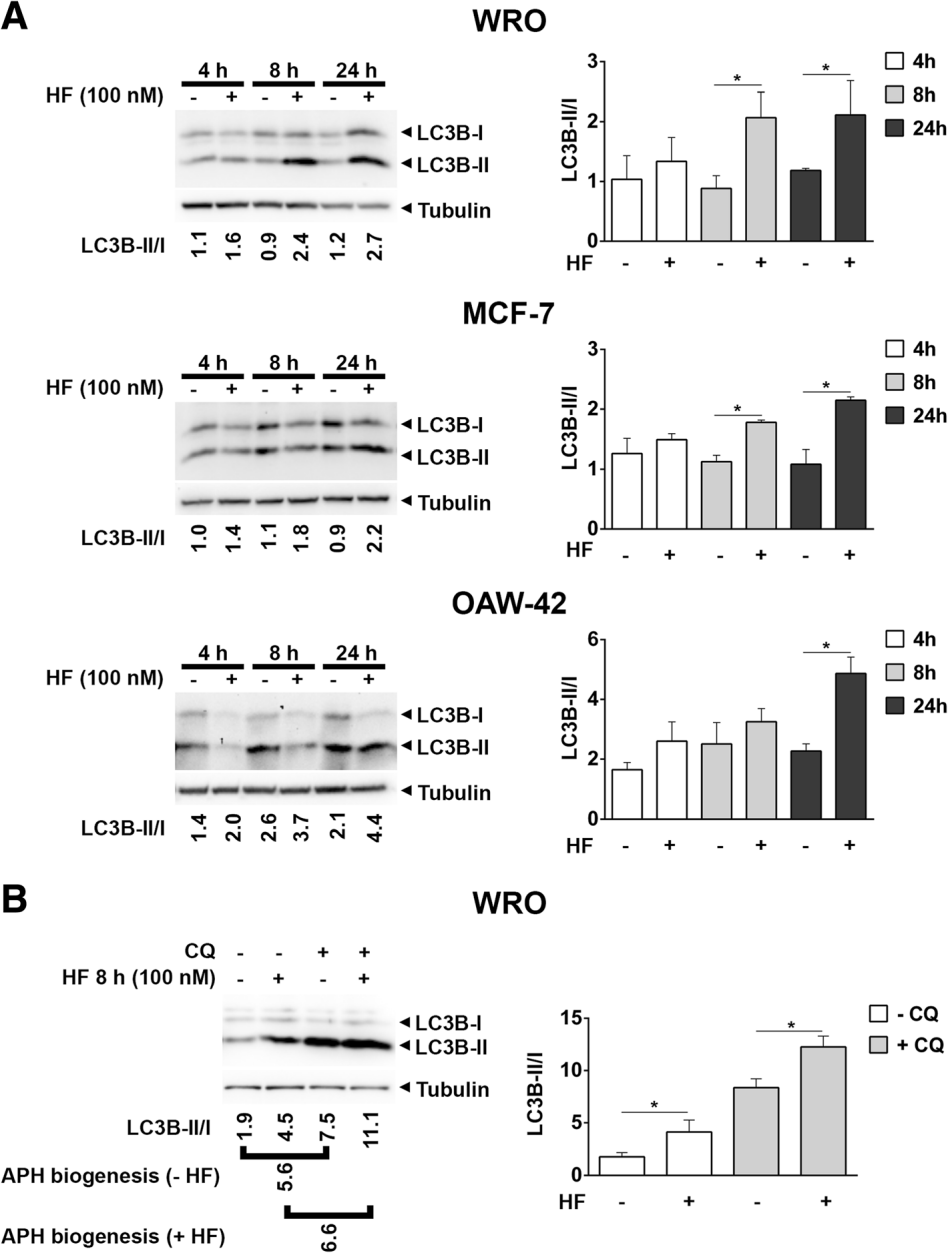
Halofuginone induces autophagy. **a** Cells were exposed to halofuginone (HF) as in Fig. 1 and LC3B protein levels were assessed by immunoblotting. As loading control, filters were stripped and probed with ant β-Tubulin as loading control. Representative immunoblots are shown along with LC3B-II/I band intensity ratios as index of autophagosome formation. Histograms show mean (±SD) LC3B-II/I band intensity ratios of three different experiments. Statistically significant differences between LC3B-II/I ratios after to before HF are shown (*, *p* ≤ 0.05). **b** WRO cells were exposed to halofuginone (HF) for 8 h in the presence or in the absence of 30 μM chloroquine (CQ) and LC3B protein levels were assessed by immunoblotting. As loading control, filters were stripped and probed with anti β-Tubulin as loading control. Representative immunoblots are shown along with LC3B-II/I band intensity ratios as index of autophagosome formation. Relative amount of newly formed autophagosomes is expressed as LC3B-II/I ratios difference in the presence or absence of CQ (APH biogenesis). Histograms show mean (±SD) LC3B-II/I band intensity ratios of 3 different experiments. Statistically significant differences between LC3B-II/I ratios after to before HF are shown (*, *p* ≤ 0.05)

A typical pattern of LC3B-I and of LC3B-II present in the cells is shown in Fig. 2a. The conversion of LC3B-I to LC3B-II, which is indicative of autophagosome formation [20], increased in all cell lines upon exposure to HF (Fig. 2a, LC3B-II/I ratio). Like for eIF2α phosphorylation, autophagy was differently modulated in the cell lines tested during the incubation time, likely reflecting the different genetic and proteome background and intracellular pool of amino acids. A significant increase of LC3B-II/I ratio was observed by 8 h in WRO and MCF7 cells, and by 24 h in OAW-42, indicating an increase in the autophagosome formation following exposure to HF. In the case of WRO cells, we also noted that the chronic (24 h) exposure to HF causes some 45% cell detachment and apoptosis (data not shown). From now on, we chose the WRO cells as representative to investigate more in depth the mechanisms linking the AAR with autophagy, limiting the exposure to HF at 8 h to avoid mis-interpretation due to cell toxicity.

Next, we assessed the accumulation of LCB-II in the presence or absence of 30 μM chloroquine (CQ) by immunoblotting to confirm that basal autophagy is up-regulated following HF. Chloroquine alkalinizes the acidic compartments, preventing the autophagosome-lysosome fusion and impairing the degradation of autophagosomes and of their content [19, 21]. Assuming the net conversion of LC3B-I into LC3B-II (ratio LC3B-II/I) in the presence vs absence of CQ as the relative amount of newly formed autophagosomes (APH biogenesis), basal autophagy was clearly up-regulated following 8 h of exposure to HF (Fig. 2b).

**Fig. 3:**
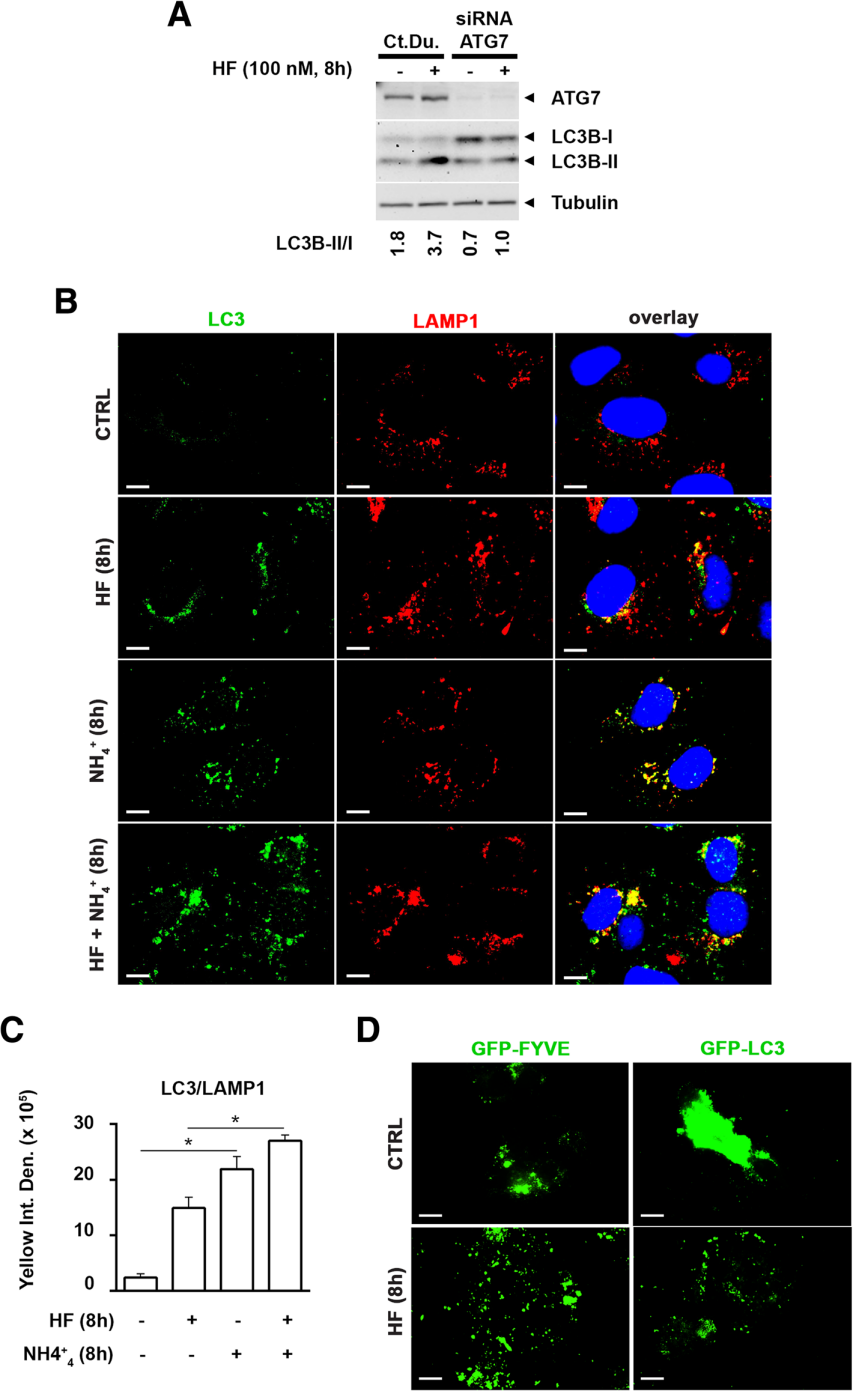
Halofuginone induces autophagosome formation and eventually interferes with its fusion with lysosomes. **a** WRO cells were transiently transfected with ATG7 siRNA or control duplex (Ct. Du.) siRNA. After 36 h, the cells were exposed to 100 nM halofuginone (HF) for 8 h. The expression of ATG7, LC3B and β-Tubulin was analyzed by immunoblotting of cell homogenates. Representative immunoblots are shown along with LC3B-II/I band intensity ratios as index of autophagosome formation. **b** WRO cells plated on coverslips were treated with 100 nM halofuginone (HF) in the presence or absence of 10 mM ammonium chloride (NH_4_^+^). After 8 h the cells were fixed, processed for LC3 (green) and LAMP-1 (red) immunostaining and imaged by fluorescence microscopy. Nuclei were stained with DAPI. Scale bars: 10 μm. **c** Bars indicate the average yellow fluorescence intensity density of immunofluorescences shown in **b**. Data are from 5 different images for each condition. Error bars: standard deviation. Statistically significant differences between fluorescence intensity densities before and after NH_4_^+^ are shown (*, *p* ≤ 0.05). The images shown are representative of four separate experiments. **d** WRO cells were plated on coverslips and transiently transfected with vectors expressing GFP-FYVE or GFP-LC3. After 36 h, the cells were exposed to 100 nM halofuginone (HF) for 8 h. Following HF, cells were imaged by fluorescence microscopy. Scale bars: 10 μm

We then assessed LC3B-II in WRO cells transfected with the siRNA targeting the essential autophagy protein ATG7 or with the control RNA duplex to confirm that the increase of LC3B-II following HF results from the induction of autophagy. LC3B-II/I ratio increased upon incubation with HF as expected, and such increase was annulled when ATG7 was silenced (Fig. 3a).

We investigate the induction of autophagy also by LC3B immunofluorescence in WRO cells exposed for 8 h to HF, NH_4_^+^, or both (Fig. 3b, c). Like chloroquine, NH_4_^+^ prevents the autophagosome-lysosome fusion, and impairs the degradation of autophagosomes and of their content including the LC3B-II bound to the inner membrane of the autolysosomes. Cells were stained for LC3B that results in a punctate fluorescence corresponding to the lipidated LC3B-II either bound to the autophagosomes or autolysosomes, and for LAMP-1 (*lysosomal associated protein-1*) that labels both endosomes/lysosomes and autolysosomes. In WRO cells exposed to HF an increase of LC3B puncta, indicative of lipidated LC3B either bound to the autophagosomes (in green) or autolysosomes (in yellow) is clearly evident and suggests the up-regulation of autophagy. Exposure to NH_4_^+^ alone led to the accumulation of vesicles that reflect the basal level of autophagy. The vesicle accumulating following NH_4_^+^ are mainly autolysosomes (in yellow), which result from the impairment of LC3B degradation in the autolysosome, together with few autophagosomes (in green), which result from the impairment of the autophagosome-lysosomes fusion. When the cells were exposed to HF in the presence of NH_4_^+^ the amount of LC3B-II only labeled (green) vesicles was even greater, indicating that more autophagosomes not fused with lysosomes were accumulating in the cells. Moreover, the increase in yellow-labeled vesicles following NH_4_^+^ was greater in cells not exposed to HF, indicating a suboptimal fusion between autophagosome and lysosomes in HF-treated cells. We confirmed the induction of autophagy by HF in WRO cells transiently expressing either GFP-FYVE or GFP-LC3. Transfected cells showed a punctate GFP fluorescence following exposure to HF that indicates the induction of autophagy and formation of autophagosomes (Fig. 3d).

Taken together, the above data support the view that HF has a dual-effect on the autophagy system: initially it induces the formation of autophagosomes, and later it slows-down their fusion with lysosomes and their degradation.

### Halofuginone, not amino acid starvation, allows protein synthesis along with autophagy

We investigated on the functional relationship between autophagy, AAR and global protein synthesis in the cells exposed to HF. In a first set of experiments, we included the conditions of amino acid and serum starvation (EBSS), which is known to induce both AAR and autophagy, and of mRNA translation inhibition by cycloheximide (CHX), which is known to block the elongation step.

**Fig. 4:**
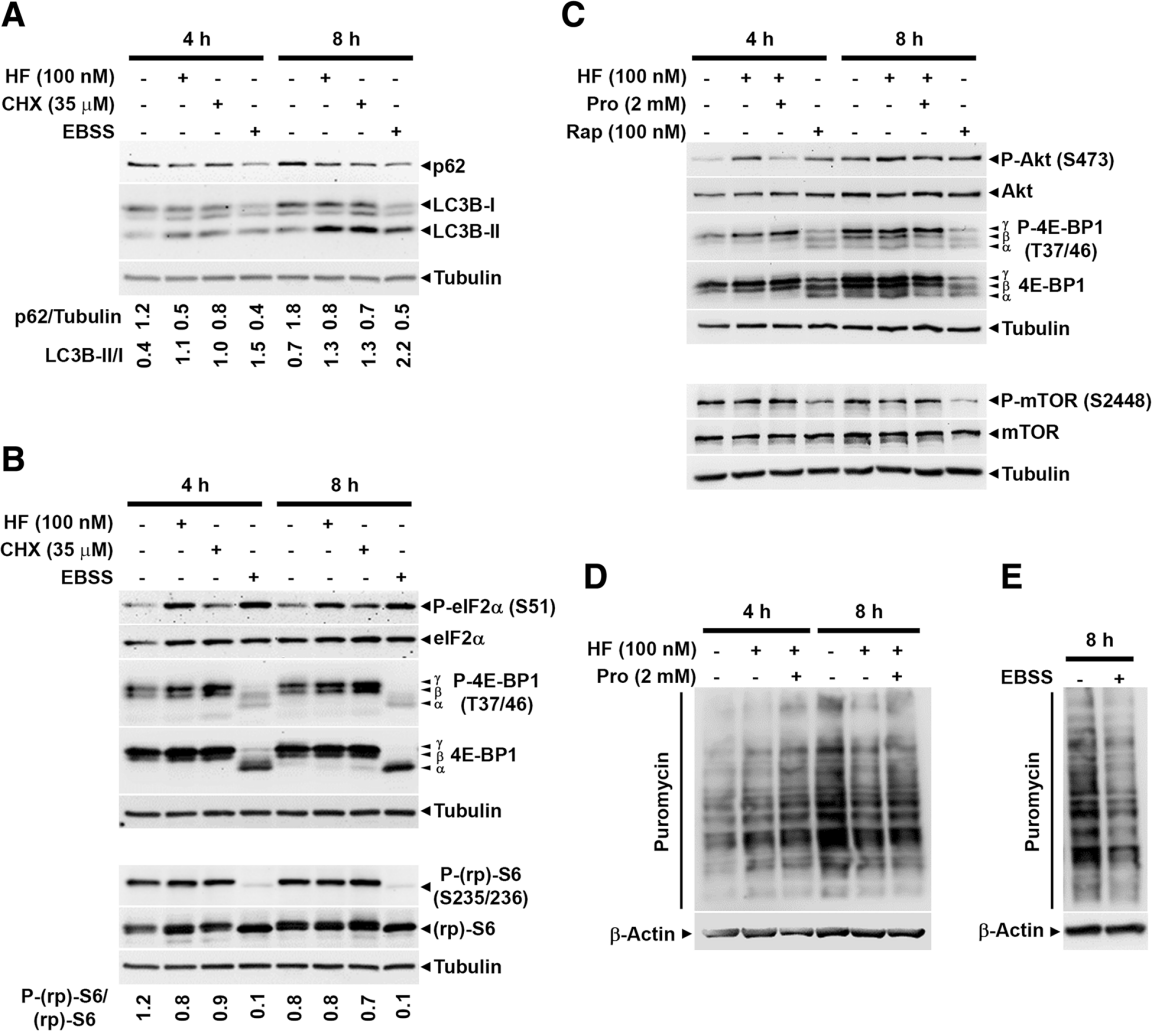
Halofuginone and amino acid starvation both induce AAR yet only the former still allows mTOR-dependent protein synthesis. **a**, **b** WRO cells were exposed for 4 and 8 h to 100 nM halofuginone (HF), 35 μM cycloheximide (CHX) or amino acid/serum growth factors deprivation (EBSS). Thereafter, the cell homogenates were assessed for markers of autophagy (LC3B, p62) (panel **a**), of AAR (P-eIF2α, phosphorylated in Ser 51) (panel **b**), and mTOR ability to promote protein synthesis (P-S6, phosphorylated in Ser 235/236, and P-4E-BP1, phosphorylated in Thr 37/46) (panel **b**). Filters were, then stripped and probed with antibodies for total eIF2α or S6 or 4E-BP1 or β-Tubulin, as indicated. Densitometry analysis of the protein bands for p62/Tubulin, LC3B-II/I and P-(rp)-S6/(rp)-S6 is shown. (**c**) WRO cells were treated with 100 nM HF, in standard medium supplemented or not with 2 mM proline (PRO), or with 100 nM rapamycin (Rap) for the time indicated. The expression of total and phosphorylated (Ser 473) Akt, total and phosphorylated (Thr 37/46) 4E-BP1, and total and phosphorylated (Ser 2448) mTOR were assessed by immunoblotting. Filters were stripped and probed with anti-β-Tubulin as loading control. **d**, **e** WRO cells were exposed for 4 and 8 h to 100 nM HF in presence or absence of 2 mM proline (PRO) (panel **d**) or incubated for 8 h in EBSS (panel **e**) and the presence of puromycin incorporated in neosynthesized proteins was revealed by immunoblotting of cell homogenates. Filters were stripped and probed with anti-β-Actin as loading control. Blots shown in this Figure are representative of three independent experiments with reproducible data

The three treatments induced autophagy, as monitored by the conversion of LC3B-I into LC3B-II (Fig. 4a). As an additional marker monitoring the autophagy flux, we determined the actual level in the cells of *sequestosome-1* (p62/SQSTM1), which tags the autophagy substrates and is degraded along with them [19]. Following HF, p62 levels decreased of about 50 to 70% compared to the control at 8 h (Fig. 4a). Based on p62 level, it is evident that the three treatments also stimulated the autophagosome degradation, besides inducing their biogenesis, being both these effects more prominent in EBSS condition.

Under the same experimental conditions, we assayed the signaling pathways governing the AAR and protein synthesis. As shown in Fig. 4b, both HF and EBSS promptly and strongly induced the phosphorylation of eIF2α, which slightly declined by 8 h, while on opposite it increased by 8 h in CHX-treated cells. These data indicate that the three treatments effectively induced an AAR. Then, we assayed the signaling pathways downstream mTORC1 governing protein synthesis. 4E-BP1 is the most studied and ubiquitously expressed member of a family of eIF4E binding proteins. Phosphorylation of 4E-BP1 at multiple sites (primed at Thr 37/46) reduces its affinity for eIF4E that becomes free and available to complex with eIF4G thus allowing the initiation of cap-dependent translation. The level of phosphorylation identifies three bands with different migratory rate, respectively named from top γ (the most phosphorylated), β (intermediate) and α (the least phosphorylated). Data in Fig. 4b show that HF and CHX maintain the hyper-phosphorylation of 4E-BP1 (indicated by the prominence of the γ band) while in EBSS is detectable only the hypo-phosphorylated isoform (α band). Next, we looked at the phosphorylation of (rp)S6, which correlates with the translational rate. We found that S6 was phosphorylated at any time in the cells incubated with HF while it was completely de-phosphorylated in the cells incubated in EBSS (Fig. 4b). S6 was phosphorylated also in the cells exposed to CHX, in which elongation of mRNA translation is blocked.

mTOR is the catalytic subunit of two distinct complexes, mTORC1 and mTORC2, that differ for composition, functionality and substrates. mTORC1 regulates, among others, the protein synthesis and autophagy processes, while mTORC2 phosphorylates, among other substrates, AKT at Serine 473. To better understand the effect of HF on mTOR-dependent protein synthesis we incubated the cells in the presence of HF with or without an excess of free proline. Rapamycin was also included in this experiment as an inhibitor of mTORC1 activity. Rapamycin effectively switched off the activity of mTOR as indicated by phosphorylation of mTOR (S2448) itself and of its downstream 4E-BP1 (Fig. 4c). HF confirmed to maintain hyper-phosphorylated 4E-BP1, while slightly reducing the phosphorylation of mTOR that was however rescued by the excess of proline (Fig. 4c). Interestingly, HF stimulated the activity of mTORC2, as testified by S473 phosphorylation of AKT, an effect that was totally reversed by proline, while Rapamycin did not affect mTORC2 activity as expected (Fig. 4c). Finally, we assessed global protein synthesis in these conditions through the puromycin incorporation assay. Data in Fig. 4d-e suggest that HF does not impair protein translation (or it does to a very little extent) while EBSS largely impairs it. Taken together, these data indicate that HF induces AAR while still allowing protein synthesis despite some inhibition of mTOR activity. In contrast, EBSS induces AAR along with total inhibition of mTOR-dependent protein synthesis.

### Halofuginone effectively links the proline-deficient AAR to autophagy

**Fig. 5:**
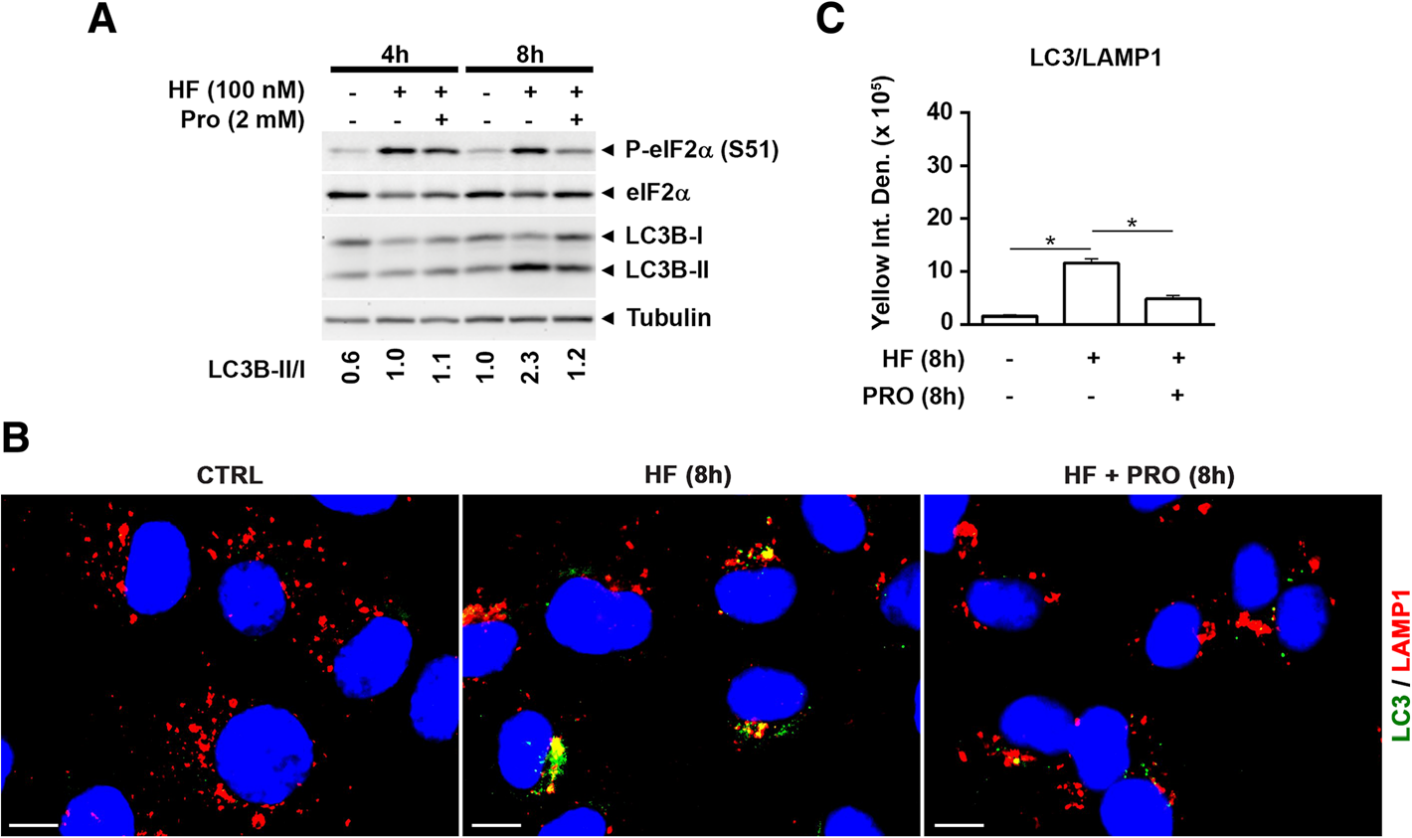
Excess of proline rescues the induction of AAR and of autophagy by halofuginone. **a** WRO cells were treated with 100 nM halofuginone (HF) in standard medium supplemented or not with 2 mM proline (PRO) as indicated. A representative immunoblotting of P-eIF2α versus total eIF2α (marker of AAR) and of LC3B (marker of autophagy) is shown. Densitometry analysis of the protein bands was performed and the LC3B-II/ I band density ratios are shown. A similar pattern of protein expression was observed in two other separate experiments. **b** WRO cells were plated on coverslips and treated as in (**a**). After 8 h the cells were fixed, processed for LC3 (green) and LAMP-1 (red) immunostaining and imaged by fluorescence microscopy. Nuclei were stained with DAPI. Scale bars: 10 μm. **c** Bars indicate the average yellow fluorescence intensity density of immunofluorescences shown in (**b**). Data are from five different images for each condition. Error bars: standard deviation. Statistically significant differences between fluorescence intensity densities after to before HF, or after to before PRO in the presence of HF are shown (*, *p* ≤ 0.05)

In theory, autophagy and AAR could be two un-related processes contemporarily induced by HF. The AAR induced by HF follows the inhibition of prolyl-tRNA synthetase activity and can be reverted by extra supplementation of proline [14]. We exploited this fact to prove definitively the ability of HF to link AAR and autophagy. The supplementation of standard culture medium with 2 mM proline was sufficient to rescue the activation of AAR pathway by HF, as indicated by the restoration of the steady state levels of eIF2α phosphorylation at 8 h (Fig. 5a). At the same time, proline supplementation almost completely abrogated the induction of autophagy by HF, as indicated by the level of LC3B protein (Fig. 5a) and by the relative amounts of LC3B-positive vesicles (green- and yellow-stained) (Fig. 5b, c) in the cells. Interestingly, extra supplementation with the amino acid leucine was unable to rescue the AAR induced by HF, demonstrating the specific action of this drug in mimicking the starvation by proline (data not shown).

From these data, we conclude that proline starvation, as mimicked by HF, links the AAR with autophagy.

### Halofuginone induces the detachment from lysosomes and subsequent proteasome-mediated degradation of mTOR

**Fig. 6:**
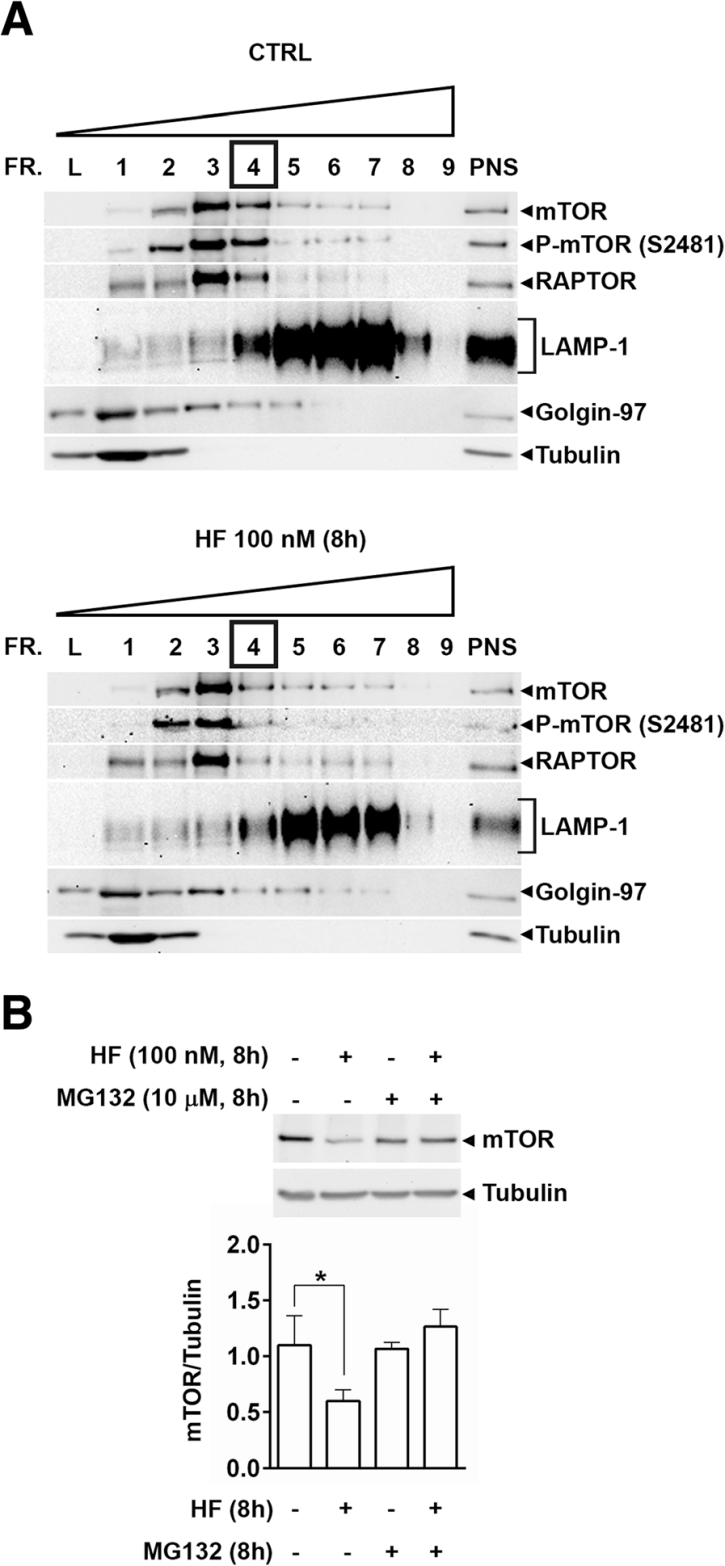
Halofuginone induces the detachment of mTORC1 from the lysosomes and partial proteasome degradation of mTOR. **a** WRO cells were exposed or not to 100 nM halofuginone for 8 h and subcellular fractions were separated by discontinuous sucrose gradient from 15 to 55% sucrose density. Nine fractions were collected and characterized by immunoblotting with the indicated antibodies. A strong reduction in the content of mTOR and RAPTOR proteins is clearly appreciable in fraction 4 of WRO cells treated with HF. A similar trend was observed in two other independent experiments and with diverse sucrose gradient. Post-nuclear supernatant (PNS) and gradient loading fraction (L) were loaded as controls. FR.N.: fraction number. **b** WRO cells were exposed or not to 100 nM of halofuginone (HF), or 10 μM of the proteasome inhibitor MG132, or both. After 8 h, the cells were collected and homogenates processed for immunoblotting to assess mTOR protein level. As loading control, the filter was stripped and probed with anti β-Tubulin antibody. The pattern of protein expression shown was reproduced in three separate experiments. Densitometry of the protein bands was performed and average mTOR/Tubulin ratios are shown in the histogram graph. Error bars: standard deviation. Statistically significant differences between mTOR protein levels are shown (*, *p* ≤ 0.05)

In the presence of amino acids, active mTORC1 is recruited on the membrane of lysosomes and phosphorylates its substrates to promote protein synthesis and to inhibit autophagy. Conversely, amino acid starvation releases mTORC1 from the lysosome membrane and this results in the activation of the ULK1 complex and of the autophagy interactome [3, 22, 23]. We reasoned that to raise up basal autophagy, HF would cause the detachment of mTORC1 from the lysosomes. Thus, we checked the abundance of mTORC1 in the lysosome fraction of WRO cells treated for 8 h with HF. Subcellular fractions were separated by discontinuous sucrose gradient and characterized by western blotting for organelle enrichment using LAMP-1, as a marker of lysosome, and Golgin-97, as a marker of the Golgi Complex. Tubulin, a component of the cytoskeleton, was used as a marker of the cytosolic fraction. RAPTOR, a unique component of mTORC1, was used to discriminate mTORC1 from mTORC2 [24, 25]. A typical pattern of the subcellular localization of mTOR (both total and phosphorylated at Ser 2481) and of RAPTOR is shown in Fig. 6a. This experiment evidenced that upon treatment with HF the abundance of mTOR and of phospho-mTOR is greatly reduced in the lighter lysosome-containing fraction 4 (possibly corresponding to unloaded small lysosomes). Concurrently, RAPTOR protein level decreased only in the same fraction in which mTOR also decreased upon HF treatment (i.e., fraction 4). Noteworthy, in untreated cells, fraction 4 is the one, among all the LAMP-1 positive fractions [4 to 8], containing the largest amount of mTOR (both total and phosphorylated at Ser 2481) and of RAPTOR (Fig. 6a). Interestingly, fractions 2 and 3, enriched in cytoplasmic (Tubulin) and Golgi-associated (Golgin-97) proteins and poor of lysosomes (the relative amount of LAMP-1 in these fractions is negligible), are positive for mTOR and RAPTOR. It is to be noted that the relative amount of mTORC1 proteins in these fractions was not increased upon HF treatment (Fig. 6a).

Thus, upon HF treatment the mTORC1 is no more associated with (small) lysosomes though apparently it does not relocate to the lighter subcellular fractions.

Indeed, we found that the absolute amount of mTOR in cell homogenate and in the cytoplasmic fraction obtained by differential centrifugation was reduced in the cells treated with HF (data not shown). We thus hypothesized that HF could induce the proteolysis of mTOR soon after provoking its detachment from the lysosome. The proteasome would be the candidate for performing such proteolysis. We therefore assessed the protein level of mTOR in WRO cells exposed to HF for 8 h in the absence or the presence of the proteasome inhibitor MG132. As shown in Fig. 6b, the cellular amount of mTOR protein was lower in HF-treated than in control cells, and the concomitant treatment with MG132 prevented the loss of mTOR induced by HF. These data indicate that upon treatment with HF mTOR is indeed degraded, though not completely, by the proteasome.

### The excess of proline prevents the detachment of mTOR from lysosomes and its degradation induced by halofuginone

**Fig. 7:**
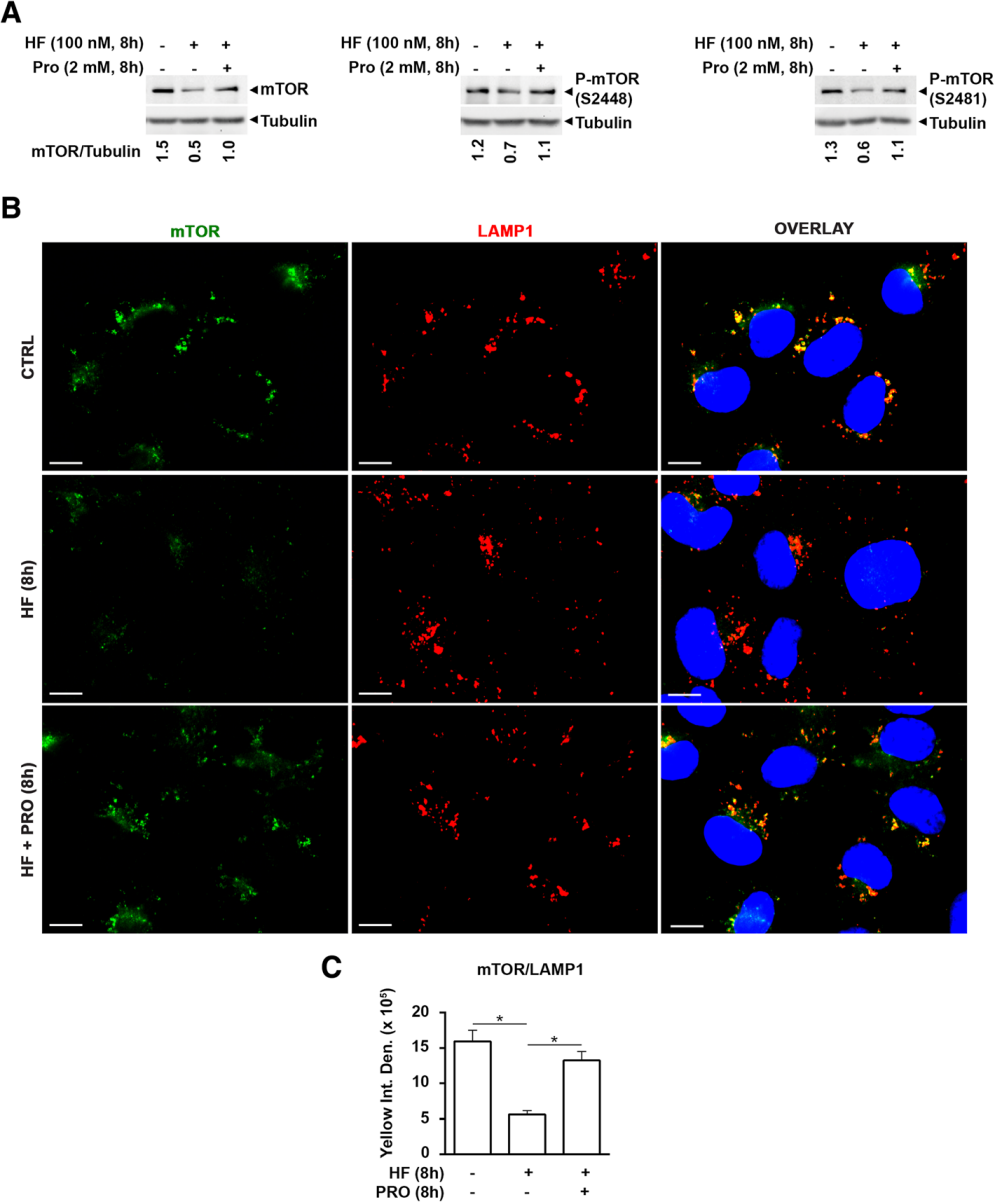
AAR pathway induced by halofuginone triggers mTOR degradation. **a** WRO cells were exposed or not to 100 nM halofuginone (HF) in the presence or in the absence of additional 2 mM proline (PRO). After 8 h, the cell homogenates were subjected to immunoblotting to assess total mTOR protein level and its phosphorylation *status* (at Ser 2448 and Ser 2481). The same set of samples was loaded in three different gels in order to avoid multiple stripping and re-probing of the same filter. Each filter was probed with anti β-Tubulin antibody as loading control. The pattern of protein expression shown was reproduced in three separate experiments. Densitometry of the protein bands was performed and mTOR/Tubulin ratios are included. **b** WRO cells adherent to coverslips and treated as in (**a**) were fixed, processed for mTOR (green) and LAMP-1 (red) immunostaining and imaged by confocal fluorescence microscopy. Nuclei were stained with DAPI. Scale bars: 10 μm. The images shown are representative of three separate experiments. **c** Bars indicate the average yellow fluorescence intensity density of immunofluorescences shown in (**b**). Data are from five different images for each condition. Error bars: standard deviation. Statistically significant differences between fluorescence intensity densities after to before HF, or after to before PRO in the presence of HF are shown (*, *p* ≤ 0.05)

We have previously shown that the extra supplementation of proline could rescue the effects of HF in inducing the AAR and autophagy. At this point, it was mandatory to close the circle and prove that mTOR links the AAR with autophagy, and that its degradation induced by HF is indeed the mechanism for inducing autophagy. Thus, the level of total and phosphorylated (active) mTOR protein was determined by western blotting in cells exposed to HF in the absence or presence of additional proline. As shown in Fig. 7a, proline supplementation rescued the reduction of total mTOR protein levels provoked by HF. Phosphorylated mTOR at Ser 2448 and Ser 2481 were also decreased in HF-treated cells, likely reflecting the decreased amount of the total protein rather than a specific impairment of mTOR phosphorylation. Again, proline supplementation rescued the levels of both phosphorylated isoforms of mTOR in the cells co-treated with HF. We have hypothesized that upon HF treatment mTOR is degraded soon after its detachment from the lysosomes. We tested this hypothesis by looking at the lysosomal localization of mTOR in the cells treated with HF in the absence or presence of extra proline. To this end, LAMP-1 and mTOR were identified by immunofluorescence staining and their co-localization assessed by fluorescence microscopy. The images in Fig. 7b and c show a general reduction of labeled mTOR (in green) and more specifically of mTOR co-stained with LAMP-1 (in yellow) in the cells treated with HF, which are consistent with the detachment from lysosomes and degradation of the protein. Noteworthy, proline supplementation rescued the subcellular localization of mTOR at the level of the lysosomes observed in not treated cells, indicating that the excess of proline could counteract the effects of HF on mTOR localization and degradation.

### Chronic treatment with halofuginone induces the nuclear relocation of the autophagy transcription factor TFEB

**Fig. 8:**
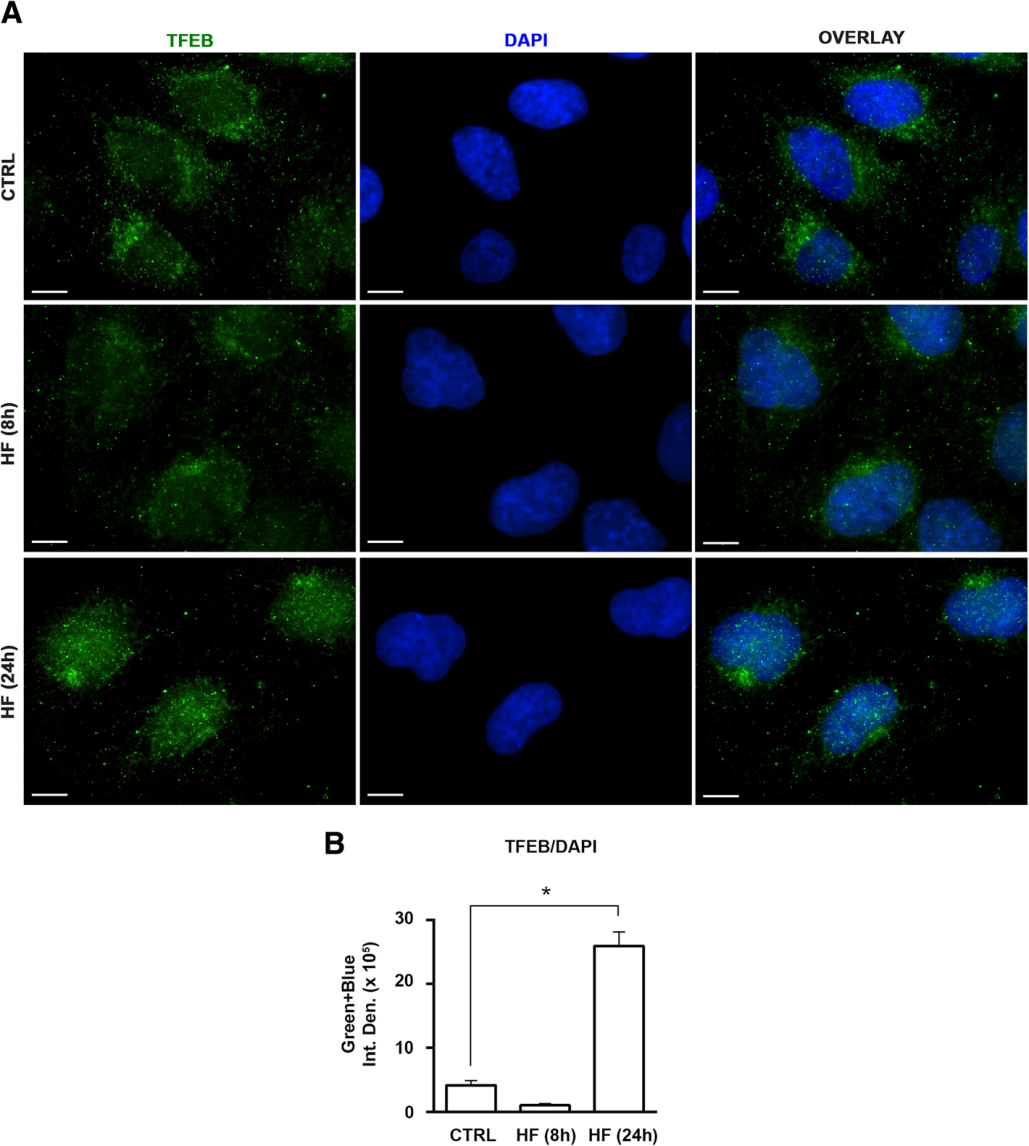
TFEB relocates into the nucleus upon chronic induction of AAR by HF. **a** WRO cells plated on coverslips were treated or not with 100 nM halofuginone. After 8 and 24 h, the cells were fixed, processed for TFEB (green) immunostaining and imaged by confocal fluorescence microscopy. Nuclei were stained with DAPI. Scale bars: 10 μm. The images shown are representative of three separate experiments. **b** Bars indicate the average green + blue fluorescence intensity density of immunofluorescences shown in (**a**). Data are from five different images for each condition. Error bars: standard deviation. Statistically significant difference between fluorescence intensity densities after to before HF is shown (*, *p* ≤ 0.05)

In nutrient rich conditions, lysosomal-associated mTORC1 phosphorylates the *transcription factor EB* (TFEB). As a result, TFEB remains sequestered in the cytoplasm and is transcriptionally inactive. Upon activation, as in the case of amino acid starvation, mTORC1 is released from the lysosomes and TFEB, not phosphorylated by mTORC1, relocates to the nucleus and can initiate the transcription of ATG genes [26]. We assessed by immunofluorescence the subcellular localization of TFEB in WRO cells after exposure to HF. Partial relocation of TFEB from the cytosol to the nucleus was clearly detectable when the cells were treated with HF for 24 h (Fig. 8a, b).

## Discussion

When subjected to nutrient starvation the cell activates an array of protective responses to face the metabolic stress. Yet, if chronically deprived of nutrient and oxygen, the cell eventually succumbs despite of, or because of, the prolonged activation of these metabolic stress responses. In particular, amino acid starvation triggers two main stress responses: the AAR, which inhibits protein synthesis, and autophagy, which degrades the redundant self-structures in an attempt to provide the substrates needed for survival. This study was designed to dissect the cross-talk between these two metabolic stress pathways triggered in response to amino acid starvation. The AAR is a pathway finalized to inhibit protein synthesis when amino acids are not available. The first step in this pathway is the phosphorylation of eIF2α at Serine 51 [6]. In parallel, ATF4, a transcription factor of the *cAMP response element binding* protein (CREB), is synthesized [27]. In turn, ATF4 promotes the transcription of genes involved in biosynthesis, transport and metabolism of amino acids, including the aminoacyl-tRNA synthetases. Importantly, the eIF2α-ATF4 pathway has been linked to the stress-induced expression of ATG genes [28]. Autophagy is per se triggered by amino acid starvation via the mTORC1 pathway. A complex regulatory network modulates Rags activity, and in turn mTORC1, by sensing amino acids levels in the cytosol or inside the LEL lumen. Cytosolic proteins have been reported to be involved in sensing leucine (*leucyl-tRNA synthetase*, LRS; Sestrin 2) [29, 30], or arginine (*cellular Arginine sensor for mTORC1*, CASTOR1) [31, 32], or amino acids (*cytoslic folliculin*, FLCN; *folliculin interacting protein*, FNIP) [33] levels in the cytosol and regulate Rags activity. Transmembrane proteins have been identified to sense alanine, proline and glycine (SLC36A1, PAT1) [34], or arginine (SLC38A9, SNAT9) [35–37], or amino acids (vacuolar-adenosine triphosphatase proton pump, v-ATPase) [23] levels inside the LEL lumen and to regulate Rags. Other works support a model in which the LEL are not the only subcellular hub for amino acids-dependent mTORC1 regulation [10]. In fact, the transmembrane protein SLC36A4 (PAT4) has been reported to sense glutamine and serine levels inside the trans-Golgi network lumen and to modulate the activity of mTORC1 localized on the Golgi membranes [38].

HF has been previously reported to induce autophagy [39–41]. In nutrient-rich conditions, HF induces autophagy by inactivating mTORC1 and the resulting dephosphorylation of ULK1 at Ser 757 [42]. Here, we show that mTORC1 specifically located at the lysosome level links the AAR induced by HF with the autophagy pathway. More in depth, our data show that the induction of the AAR pathway triggers autophagy by promoting the detachment from the lysosomes and the proteasome degradation of mTOR. Subcellular fractionation analyses showed that it is the lysosome-associated, not the Golgi Complex-associated pool of mTOR that is degraded by the proteasome. On long term, the sustained AAR and autophagy led to the nuclear translocation of TFEB, a transcription factor of many ATG genes. In homeostatic conditions, TFEB is stabilized in the cytoplasm by mTORC1-mediated phosphorylation. Thus, only when LEL-associated mTOR is extensively degraded the pool of (un-phosphorylated) TFEB can translocate into the nucleus and direct the synthesis of pro-autophagy genes.

As expected following the undocking of mTOR from the lysosomes and in line with recent works [39, 41], we observed the induction of ATG by HF in all the analyzed cell lines. On long term sustained AAR and autophagy, as induced by HF, led to apoptosis. It was previously reported that the chronic up-regulation of ATF4 leads to the transcription of DNA-damage- inducible transcript 3, which in turn promotes the transcription of pro-apoptotic genes [43]. Thus, AAR and autophagy pathways intersect at two stages of the stress response, in the early stage to coordinate the block of protein synthesis and the degradation of the intracellular pool of protein, and in the later stage to coordinate the programmed cell death. It remains to clarify whether apoptosis in HF-treated cells ensued in spite of or because of the hyper-activation of autophagy.

Here, we used HF as a strong inducer of AAR. HF mimics a reduced cellular pool of available proline by competing with it for the prolyl-tRNA synthetase active site [14]. The resulting accumulation of uncharged tRNA^PRO^ leads to the activation of the AAR pathway. AAR induced by HF reached a peak at different time of incubation in the different cell lines tested, probably reflecting the different pool of free proline available in the cell. HF indeed acted much alike CHX (an inhibitor of protein synthesis) and EBSS (a condition of amino acid and serum starvation) in inducing the AAR and autophagy pathways. The extra supplementation of proline, not of leucine (known to be a major regulator of mTOR), rescued all the phenotypic features triggered by HF, including the effects on mTOR degradation. Interestingly, HF stimulated the phosphorylation of AKT, an effect that was reversed by proline. We may speculate that HF by disrupting mTORC1 from the lysosomal membrane frees mTOR which is partly degraded by the proteasome and partly made available to complex in mTORC2. However, this hypothesis must be proven experimentally.

More importantly, we found differences between HF and amino acid starvation (EBSS) with respect to mTOR-dependent protein synthesis control. The signaling pathways governing protein synthesis and their alterations in cancer cells have been the subject of recent excellent reviews [44–46]. Briefly, in the presence of amino acid and of active PI3K-AKT, mTORC1 can phosphorylate p70S6k and 4E-BP1. P70S6k will then phosphorylate (S235/236) S6, whose level correlates with the rate of mRNA translation. Phosphorylation of 4E-BP1 reduces its affinity for eIF4F, which can then bind to eIF4G and allows the initiation of translation. We found that HF could maintain 4E-BP1 highly phosphorylated (γ isoform), while in EBSS only α-4E-BP1 (faintly phosphorylated form) was apparent. This has been associated with ongoing and repressed protein synthesis situations, respectively. In fact, while α and β isoforms (i.e., the least and intermediate phosphorylated forms) of 4E-BP1 associate with eIF4E, the appearance of the γ isoform (the most phosphorylated one) reflects the release of eIF4E [47, 48]. Notably, in EBSS the total amount of 4E-BP1 appeared reduced. It has been shown that in cancer cells hyper-phosphorylation of 4E-BP1 at multiple sites plays an important role in its stabilization and overexpression [49], which may explain the higher level of the γ isoform observed in control and HF-treated cells. We also found that in HF-treated, but not in EBSS-treated cells, S6 was phosphorylated, despite HF reduced to some extent mTOR phosphorylation. Consistent with the signaling, the puromycin incorporation assay showed that protein synthesis was largely suppressed in the cells incubated in EBSS while it was little if any affected when exposed to HF in complete medium. Thus, compared to complete starvation, HF treatment may present the advantage to stimulate the removal of potentially dangerous material by autophagy while allowing protein synthesis. In other words, HF permits the recycling of the basic components resulting from the degradation of autophagic *cargo* in new essential cellular constituents, while this process is precluded during starvation. The effects seen with HF resemble those reported for resveratrol (RV), a nutraceuticals that acts as a protein restriction mimetic [50, 51]. In a separate work, we have shown that culturing ovarian cancer cells in complete medium containing RV or in EBSS inhibited protein synthesis, as indicated by down-phosphorylation of mTOR and of its downstream target eIF4E-binding protein 1 (4E-BP1) and of S6 and concomitant hyper-phosphorylation of eIF2α, while inducing autophagy in parallel [51]. However, EBSS was more effective in inhibiting protein synthesis while RV was more effective in inducing autophagy [51]. In a recent study, Xia and colleagues have shown that HF-induced autophagy suppresses MCF-7 migration and invasion by down-regulating STMN1 and p53, indicating that HF-induced autophagy may play an important role in the anti-cancer effect of HF [41]. Intriguingly, RV too was shown to suppress the migration of ovarian cancer cells by raising the level of autophagy [52].

HF is a racemic halogenated derivate of febrifugine, an alkaloid extract of the plant Blue Evergreen Hydrangea (*Dichroa febrifuga Lour*) known for its antiprotozoal activity and used as antimalarial remedy in traditional Chinese medicine [14, 53]. The mechanism of its antimalarial property remains obscure. Our data suggest that this property is related to its effects on the autophagy-lysosome system. The lysosome is an indispensable station for the maturation cycle of the plasmodium, and drugs able to buffer the acidity in the lysosome, such as chloroquine, disrupts this cycle. Chloroquine is now known to also impair the last step of the autophagy process. Here, we found that HF initially induces autophagosome formation and later impairs the autophagosome-lysosome fusion step, much alike chloroquine. Of note, our work is the first to show a dual-effect of HF on autophagy as the other studies investigating HF-induced autophagy reported only the induction of autophagy by the HF and not an impairment of the autophagosome-lysosome fusion step [39, 41]. We hypothesize that the effect of HF on the autophagosome-lysosome fusion step may differ depending on the cell line, HF concentration and incubation time. More investigation is needed to clarify the effect of HF on the autophagosome-lysosome fusion step since in this work we focused only on short term incubation time to avoid mis-interpretation due to cell toxicity. HF elicits also a general anti-inflammatory response by inhibiting the differentiation of inflammatory Th17 cells, and this effect was clearly sue to the induction of the AAR [13–15]. HF is currently employed in clinical trials due to its therapeutic potential in fibrotic diseases and cancer [42, 54–59] (https://clinicaltrials.gov/). The data here reported strongly support the view that the therapeutic potential of HF is linked to its ability to induce autophagy, and eventually cell death, besides its modulatory effect on protein synthesis.

## Conclusions

**Fig. 9:**
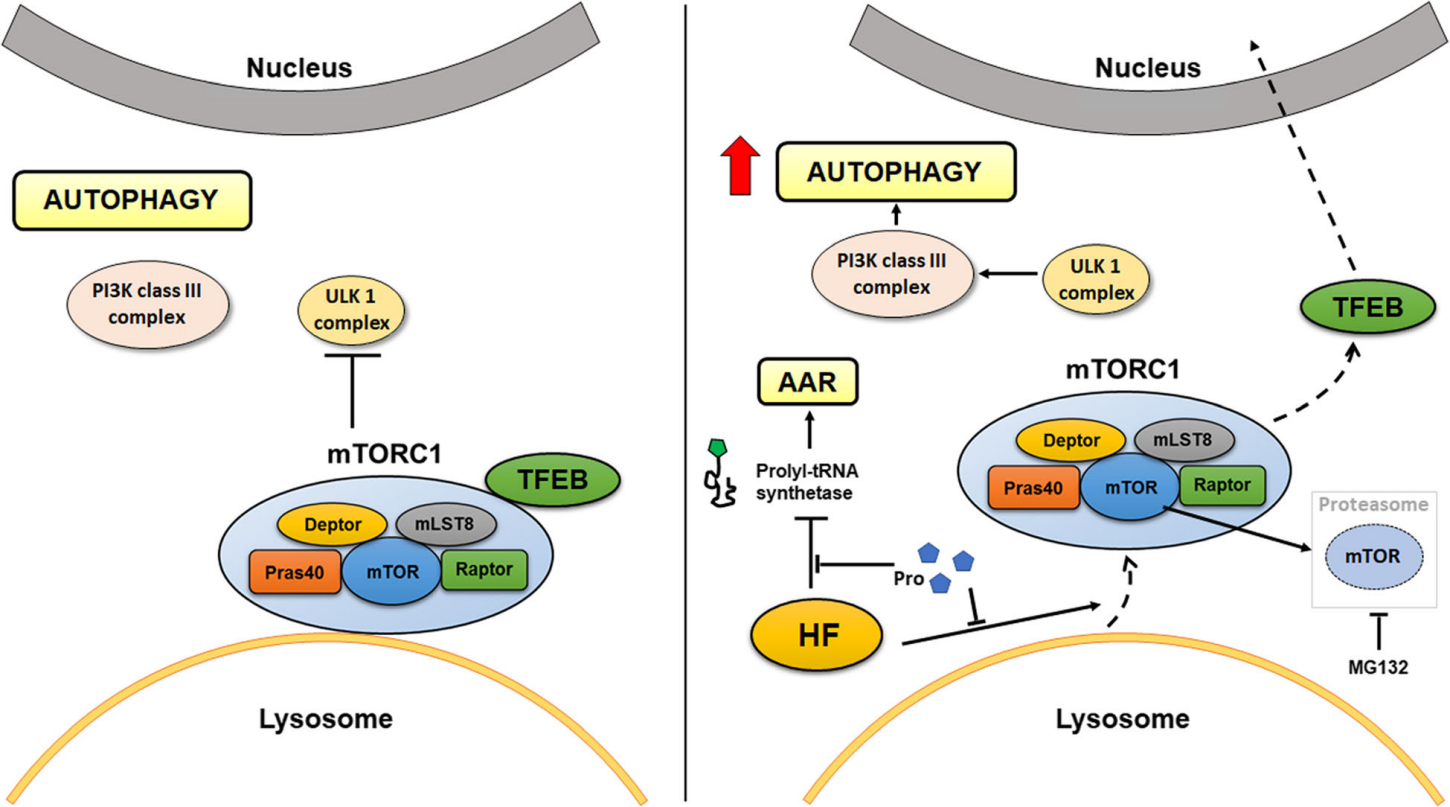
Schematic representation of the main findings of the study. Under normal conditions (left), mTORC1 is recruited on the membrane of the lysosomes and downregulates autophagy by inhibiting ULK1 complex. HF (right) triggers the Amino Acid starvation Response (AAR) by inhibiting the prolyl-tRNA synthetase and induces the detachment of mTORC1 from the lysosomes. As result, mTORC1 no longer inhibits ULK1, TFEB detaches from mTORC1 and relocates into the nucleus, and autophagy is induced. Following HF, mTOR is partially inactivated and degraded by the proteasome. Extra supplementation of proline rescues the induction of AAR, mTORC1 detachment from the lysosomes, mTOR degradation and autophagy induction

The cartoon shown in Fig. 9 summarizes the main findings reported in this study. Overall, our data demonstrate that the AAR and autophagy are mechanistically linked by mTORC1, and that the lysosome is the central hub of the cross-talk between these two metabolic stress responses. Our results also suggest that the therapeutic potential of HF is linked to its ability to trigger autophagy.

## Abbrevations

AAR: Amino Acid starvation Response
ATF4: activating transcription factor 4
ATG: autophagy
CHX: cycloheximide
CQ: chloroquine
EBSS: Earle’s Balanced Salt Solution
eIF2α: eukaryotic translation initiation factor 2α
HF: halofuginone
LAMP-1: lysosomal associated protein-1
LC3B: microtubule-associated protein 1 light chain 3 isoform B
LEL: late endosomes/lysosomes
mTOR: mammalian target of rapamycin
mTORC1: mammalian target of rapamycin (mTOR) complex 1
NH_4_^+^: ammonium chloride
p62/ SQSTM1: sequestosome-1
PRO: proline
RAPTOR: regulatory-associated protein of mTOR
S6: ribosomal protein S6
TFEB: transcription factor EB
